# Overcoming the Challenges of High Quality RNA Extraction from Core Needle Biopsy

**DOI:** 10.3390/biom11050621

**Published:** 2021-04-22

**Authors:** Hanne Locy, Rohann J.M. Correa, Dorien Autaers, Ann Schiettecatte, Jan Jonckheere, Wim Waelput, Louise Cras, Stefanie Brock, Stefaan Verhulst, Keith Kwan, Marian Vanhoeij, Kris Thielemans, Karine Breckpot

**Affiliations:** 1 Laboratory for Molecular and Cellular Therapy, Department of Biomedical Sciences, Vrije Universiteit Brussel (VUB), Laarbeeklaan 103/E, B-1090 Brussels, Belgium; dorien.autaers@vub.be (D.A.); kris.thielemans@vub.be (K.T.); 2 Department of Radiation Oncology, London Regional Cancer Program, London Health Sciences Centre, 800 Commisioners Road East, London, ON N6A 4G5, Canada; rohann.correa@lhsc.on.ca; 3 Department of Radiology, Universitair Ziekenhuis Brussel (UZ Brussel), Laarbeeklaan 101, B-1090 Brussels, Belgium; ann.schiettecatte@uzbrussel.be (A.S.); jan.jonckheere@uzbrussel.be (J.J.); 4 Department of Anatomo-Pathology, UZ Brussel, Laarbeeklaan 101, B-1090 Brussels, Belgium; wim.waelput@uzbrussel.be (W.W.); louise.cras@uzbrussel.be (L.C.); stefanie.brock@uzbrussel.be (S.B.); 5 Liver Cell Biology Research Group, Department of Biomedical Sciences, VUB, Laarbeeklaan 103/D, B-1090 Brussels, Belgium; stefaan.verhulst@vub.be; 6 Department of Pathology and Laboratory Medicine, Western University, London, ON N6A 3K7, Canada; keith.kwan@lhsc.on.ca; 7 Department of Surgery, UZ Brussel, Laarbeeklaan 101, B-1090 Brussels, Belgium; marian.vanhoeij@uzbrussel.be

**Keywords:** breast cancer, fresh-frozen, formalin-fixed paraffin-embedded, biopsy, RNA, gene expression

## Abstract

The use of gene expression profiling (GEP) in cancer management is rising, as GEP can be used for disease classification and diagnosis, tailoring treatment to underlying genetic determinants of pharmacological response, monitoring of therapy response, and prognosis. However, the reliability of GEP heavily depends on the input of RNA in sufficient quantity and quality. This highlights the need for standard procedures to ensure best practices for RNA extraction from often small tumor biopsies with variable tissue handling. We optimized an RNA extraction protocol from fresh-frozen (FF) core needle biopsies (CNB) from breast cancer patients and from formalin-fixed paraffin-embedded (FFPE) tissue when FF CNB did not yield sufficient RNA. Methods to avoid ribonucleases andto homogenize or to deparaffinize tissues and the impact of tissue composition on RNA extraction were studied. Additionally, RNA’s compatibility with the nanoString nCounter^®^ technology was studied. This technology platform enables GEP using small RNA fragments. After optimization of the protocol, RNA of high quality and sufficient quantity was obtained from FF CNB in 92% of samples. For the remaining 8% of cases, FFPE material prepared by the pathology department was used for RNA extraction. Both resulting RNA end products are compatible with the nanoString nCounter^®^ technology.

## 1. Introduction

Gene expression profiling (GEP) has proven a valuable strategy to advance our understanding of the molecular landscape and drug resistance mechanisms of several cancer types, including breast cancer (BC) [1,2,3,4,5,6,7,8,9,10,11,12,13,14,15,16,17,18,19,20].

With respect to BC, GEP is increasingly performed in routine practice, guiding patient management [19]. GEP has been extensively used to classify BC into subtypes, identifying transcriptional signatures for estrogen receptor^+^ (ER^+^, luminal), human epidermal growth factor receptor 2^+^ (HER2^+^) ERBB2-amplified, and ER^−^, progesterone receptor^−^ (PR^−^) and HER2^−^ (basal) BC [21]. This has resulted in the development of kits for GEP of BC samples, such as Mammaprint, Oncotype Dx Breast, Prosigna PAM50 Breast Cancer Prognostic Gene Signature Assay, Breast Cancer Index, and EndoPredict [22]. Moreover, GEP provided an overview of multi-gene interactions, as such painting a bigger picture of changes at the level of molecular pathways and networks. As a result, more detailed BC molecular subclasses have been defined. These are associated with therapy outcome [23,24]. Signatures of immune-related genes (IRGs) are also being explored in BC as a strategy to identify patients at risk of succumbing to the disease [25].

A major challenge to GEP is the sampling and preparation of biological material, i.e., the RNA required for analysis [26]. In turn, the RNA quantity and quality depend on the sample type. Formalin-fixed paraffin-embedded (FFPE) tumor samples are routinely prepared for diagnostic purposes and long-term storage. Protocols to extract nucleic acids from FFPE samples have been described as well, and core needle biopsies (CNB) have already been proven to serve as an adequate and suitable sample type for downstream GEP [27,28,29,30,31]. However, RNA can be easily degraded prior to and during the process of formalin fixation [26,32]. Moreover, GEP based on RNA extracted from FFPE samples may be subject to variation as a result of protocols used for fixation and tissue processing, bringing the need to validate results [33]. Fresh-frozen (FF) CNB that are prepared for biobanking represent an alternative source of samples. However, extracting RNA from these samples can be challenging as well, due to different steps in the protocol that have an impact on the RNA yield, purity, and integrity, including tissue disruption (homogenization) and avoidance of ribonuclease (RNase) activity. Several studies have shown that RNA extracted from FFPE samples consists of smaller fragments than RNA extracted from FF tissue [34,35].

We set up a protocol for RNA extraction from FF CNB BC samples. This protocol is made publicly available through this publication and pays attention to tissue homogenization and avoidance of RNases. We showed that the extraction of high-yield and high-quality RNA was feasible for >90% of FF CNB samples and that FFPE samples served as an adequate back-up source for RNA extraction in cases where RNA extraction failed.

## 2. Materials and Methods

### 2.1. Tumor Samples

Fresh core needle tumor samples (pancreas cancer, *n* = 1; cancer of the vulva, *n* = 1; colon carcinoma, *n* = 2, and BC, *n* = 88) and FFPE tumor samples (BC, *n* = 7) were obtained using a 16 G × 100 mm or 18 G × 100 mm core needle biopsy instrument from patients who were treated at Universitair Ziekenhuis Brussel (UZ Brussel) from December 2017 to January 2020 and who gave informed consent. Fresh CNB were collected in 50 mL tubes (Sarstedt, 62.547.254, Berchem, Belgium) containing 5 mL RNA*later*^TM^ solution (Sigma-Aldrich, R0901, Overijse, Belgium,). Samples were stored at 4 °C for a maximum of 1 month before further processing. The project followed the Helsinki Declaration and was approved by the ethics council of the UZ Brussel (2017/344 and 2017/400).

### 2.2. RNA Extraction from Core Needle Biopsies

The standard operating procedure (SOP) resulting from this work is provided in the Supplementary Materials (S1). Additional information concerning nomenclature and safety measures of different reagents used can be retrieved in Appendix A, Table A1.

#### 2.2.1. Preparation of Core Needle Biopsies for Snap Freezing

Fresh CNB were taken under ultrasound guidance and immediately transferred to a properly coded 50 mL tube (Sarstedt, 62.547.254) containing refrigerated 5 mL RNA*later*^TM^ solution (Sigma-Aldrich, R0901). To ensure emersion of the biopsy in the RNA*later*^TM^ solution, the 50 mL tube was shortly centrifuged in an Eppendorf centrifuge (Eppendorf, 5810R, Aarschot, Belgium) at 375 relative centrifugal force (rcf) for 1 min at 4 °C. All the following steps were performed on ice and in a horizontal laminar flow cabinet (Esco Global, delivered by Analis, Ghent, Belgium) that was thoroughly cleaned with RNase ZAP decontamination wipes (Invitrogen, AM9786, delivered by Thermo Fisher Scientific, Erembodegem, Belgium). These wipes were further used to clean all necessary equipment. The weight of the CNB was determined. First, a 2 mL Safe-Lock Eppendorf tube (Eppendorf, 0030121686) was labeled with the biopsy code and weighed using an analytical scale (Sartorius, CP124S). A Kimberly-Clark KimWipe disposable tissue (Merck, Z188956, Overijse, Belgium) was placed on the surface of the laminar flow cabinet. RNase-free disposable forceps (Heinz Herenz Medizinalbedarf Gmbh, 1131884, Hamburg, Germany) was placed on this tissue. The 50 mL tube containing CNB was inserted into the laminar flow cabinet on ice. Next, the CNB was transferred using the RNase-free disposable forceps to a sterile Petri dish (Falcon, 351029, delivered by VWR, Leuven, Belgium). To ensure efficient homogenization, CNB that were longer than 1 cm were cut in two with a sterile disposable scalpel (Swann-Morton, 0511, Sheffield, UK) using the same RNase-free disposable forceps to stabilize the tissue sample. CNB were transferred with the RNase-free disposable forceps to the 2 mL Safe-Lock Eppendorf tube. This tube was weighed again and the weight of the CNB was calculated by subtracting the weight of the empty tube from the weight of the biopsy-containing tube. The CNB was snap frozen by immersion of the biopsy-containing tube in liquid nitrogen. Snap-frozen samples can be stored at −80 °C.

#### 2.2.2. Homogenization and Lysis of Snap-Frozen Samples

**Figure 1:**
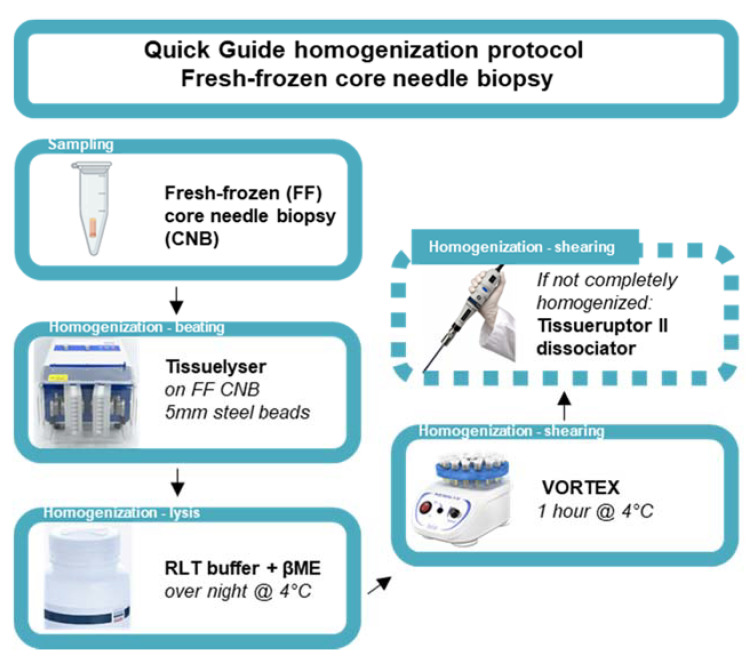
Quick guide of the homogenization protocol of fresh-frozen core needle biopsies. Schematic representation of an optimized homogenization protocol starting from ultrasound guided sampling of FF, BC CNB. Homogenization entails the disruption of BC tissue using steel beads in the Tissuelyser instrument (beating method). Next, lysis is performed using Qiagen RNA lysis buffer (RLT buffer) containing βME, followed by a one-hour vortex step at 4 °C (shearing method). If the tumor tissue is not completely homogenized, an additional dissociation step can be included using the Tissueruptor II dissociator (shearing method).

The 2 mL Safe-Lock Eppendorf tube containing the snap-frozen CNB was re-inserted into the horizontal laminar flow cabinet, and a 5 mm stainless-steel bead (Qiagen, 69989, Antwerpen, Belgium) was added using a single-bead dispenser (Qiagen, 69965). Next, the Eppendorf tube was inserted into a pre-cooled adapter (Qiagen, 11993) and subsequently inserted into the TissueLyser II instrument (Qiagen, 85300) for the first homogenization step (beating method) for 30 s at 30 Hertz. In case not all the tumor tissue was homogenized, the tumor tissue was repositioned at the bottom of the 2 mL Safe-Lock Eppendorf tube under sterile conditions using the RNase-free disposable forceps. This homogenization step was repeated for a maximum total time of 2 min (4 cycles). Next, lysis buffer consisting of RLT buffer containing 0.3718M β-mercaptoethanol (βME, Sigma-Aldrich, M6250) provided in the Qiagen RNeasy kit (Qiagen, 74104) was added according to the manufacturer’s protocol (350 µL or 600 µL lysis buffer for tissues < 20 mg or ≤30 mg, respectively). Subsequently, the 2 mL Safe-Lock Eppendorf tube containing the (homogenized) BC CNB in lysis buffer was incubated overnight at 4 °C. After 14–16 h of incubation at 4 °C, the 2 mL Eppendorf tube was transferred to a vortex instrument (Cleaver Scientific, CSLVORTEX, Warwickshire, UK) and vortexed for 1 h at 4 °C. In case the tumor tissue was not completely dissociated, the lysate and remaining tissue was transferred to a 50 mL centrifuge tube and homogenized using the TissueRuptor II dissociator until the desired degree of homogenization was obtained (Qiagen, 9002756, probes 990890). Next, the probe was detached and subsequently rinsed with lysis buffer (same volume as used for initial lysis). The lysate in the 50 mL centrifuge tube was briefly spun using the Eppendorf centrifuge. Finally, the lysate was transferred to a new 1.5 mL Eppendorf DNA LoBind tube (Sigma-Aldrich, EP0030108051) and used as starting material for subsequent total RNA extraction. Figure 1 serves as a quick guide to the homogenization protocol.

#### 2.2.3. Total RNA Extraction from Lysed Samples

Total RNA extraction was performed using the Qiagen RNeasy kit (Qiagen, 74104) according to the manufacturer’s instructions. The eluate was reloaded on the column to obtain a higher RNA yield.

### 2.3. Preparation of FFPE Tumor Specimens

The SOP resulting from this work is provided in the Supplementary Materials (S2). Additional information concerning nomenclature and safety measures of different reagents used can be retrieved from Appendix A, Table A1.

#### 2.3.1. Protocol for FFPE Preparation

FFPE tumor biopsies were generated using the Sakura instrument (Sakura, Tissue-Tek VIP^®^ 6AI Vacuum Infiltration Processor, Alphen aan den Rijn, The Netherlands). Tumor biopsies were fixed using 10% formalin for 1.5 h at 35 °C and dehydrated by immersing the tissue in different concentrations of ethanol for 4.5 h at 35 °C. Next, xylene was used as a clearing agent for 2 h at 35 °C. Finally, samples were paraffin embedded at 58 °C for 3 h.

#### 2.3.2. Ribonuclease-Free Macrodissection of FFPE Tumor Specimens

**Figure 2:**
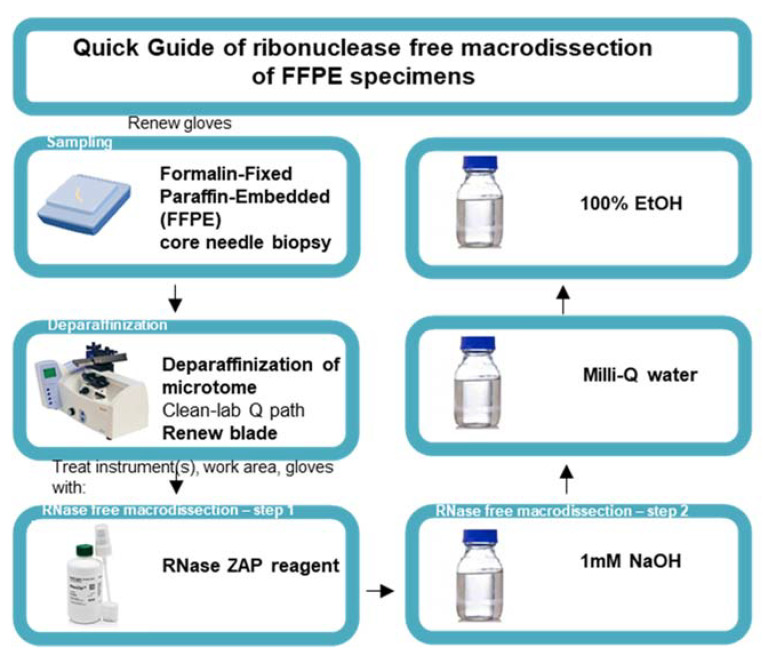
Quick guide to the ribonuclease-free macrodissection of FFPE specimens. Schematic representation of ribonuclease-free macrodissection of FFPE core needle specimens starting with the renewal of gloves for handling every new FFPE specimen, deparaffinizing the microtome, mounting a new blade, and initiating RNase-free macrodissection with the subsequent treatment of the instrument, work area, and gloves with RNase-ZAP reagent, 1 mM NaOH, Milli-Q water, and 100% ethanol (EtOH).

Steps were taken to ensure an RNase-free working station. A 100 mL solution of 1 M NaOH, 100 mL Milli-Q water, and 100 mL 100% EtOH was transferred to a Pyrex glass bottle and baked at 232 °C. The microtome device (Thermo Fisher Scientific, HM450, Erembodegem, Belgium) was prepared by removing the previous blade and by deparaffinating the microtome using deparaffinization clean lab solution (VWR, 10047400, Leuven, Belgium). Gloves were renewed and sprayed with RNase-ZAP solution (Invitrogen, AM9782). Once the gloves were air dried, the work area was cleaned using RNase-ZAP solution and forceps were placed on a Kimberly-Clark KimWipe disposable tissue. The microtome and forceps were rendered RNase free by cleaning the zones of the microtome that came in contact with the slide using RNase-ZAP wipes and KimWipes soaked with NaOH, Milli-Q water, and finally 100% EtOH. After the instrument was air dried, we mounted a new blade (Thermo Fisher Scientific, 152200) and repeated the RNase-free cleaning steps. RNase-free macrodissection was initiated on mounted FFPE blocks and the desired curls were collected in a 1.5 mL Eppendorf DNA LoBind tube. Figure 2 provides a quick guide to the macrodissection protocol.

#### 2.3.3. RNA Extraction from Collected FFPE Curls

Total RNA extraction was performed using the Qiagen RNeasy FFPE kit (Qiagen, 73504) according to the manufacturer’s instructions. Deparaffinization of the FFPE curls can be performed using the deparaffinization solution (Qiagen) or the heptane/methanol method without impact on downstream RNA applications. The eluate was reloaded on the column to obtain a higher RNA yield.

### 2.4. Quality Control of the Extracted Total RNA

A Qubit 4 Fluorometer and the Qubit RNA HS Assay Kit (Thermo Fisher Scientific, Q32852) were used to assess the RNA yield. Absorbance at 260 and 280 nm was evaluated using a NanoPhotometer Classic (Implen, delivered by Westburg, Leusden, The Netherlands). Samples were run on an Agilent 2100 bioanalyzer using the RNA 6000 nano (Agilent, 5067-1511/1512/1529, Diegem, Belgium) or pico (Agilent, 5067-1513/1514/1535, Diegem, Belgium) kit and the eukaryotic total RNA program. The bioanalyzer electropherograms were analyzed using Agilent 2100 Expert Software to determine the RNA size distribution, the RNA integrity number (RIN) value, and the DV200 values (percentage of RNA fragments with a length >200 nucleotides).

### 2.5. Functionality Control of Extracted Total RNA

RNA from FF (50 ng) and FFPE (100 ng) BC CNB were compared in terms of detection of gene expression variation using nanoString nCounter^®^ technology. Samples were hybridized according to the manufacturers’ recommendations using the nCounter^®^ Human PanCancer Immune Profiling Panel. Absolute counts were quantified by the nCounter digital analyzer (NanoString Technologies, nCounter MAX Analysis System, Seatle, United States of America). Raw counts were normalized using the RUVSeq method adjusted for nanoString nCounter^®^ gene expression analysis as described by Bhattacharya et al. [36]. Principal component analysis was performed using basic R function “prcomp.”

### 2.6. Statistical Analysis

Spearman correlations and graphical data representations were done using GraphPad Prism^®^ v8. Box plots indicate the median value and the upper and lower quartiles. Whiskers were drawn according to the minimum–maximum method, with an exception for the normalization of raw counts, for which whiskers were drawn according to the Tukey method. When applicable, significance levels were calculated using an unpaired, two-tailed Mann–Whitney test in case two groups were compared or the Kruskal–Wallis test in case of multiple groups. Statistical significance was determined as *p* < 0.05. Asterisks in the figures signify a statistically significance difference as follows: *, *p* < 0.05, **, *p* < 0.01, and ***, *p* < 0.001.

## 3. Results and Discussion

### 3.1. RNA Extraction from Fresh-Frozen Core Needle Biopsies Requires Mechanical Disruption

We optimized the procedure to isolate RNA from FF CNB in light of an ongoing phase I clinical trial in BC patients in which we perform GEP to study the immune activating capacity of intratumoral delivery of a proprietary mRNA drug, referred to as TriMix [37]. We hypothesized that FF CNB as starting material would result in higher amounts of intact RNA, since these specimens did not undergo the deleterious processing steps of FFPE block generation known to affect RNA integrity and quality [32]. As BC CNB of human origin were not abundantly available, we decided to first test different protocols for tissue homogenization or disruption with CNB available from various sources of leftover human tumor material.

We performed chemical lysis using the lysis buffer provided in the SV total RNA isolation kit of Promega on human colon, breast, and kidney tumor CNB. To that end, CNB were stored for 24 h at 4 °C in a Trizol-free lysis buffer in order to subsequently extract RNA using an easy-to-perform, non-toxic method. It was empirically determined that lysis was incomplete, as there was remaining residue. The extraction of RNA did not yield sufficiently high concentrations to quantify the RNA or assess its quality (data not shown). Therefore, we shifted towards evaluating a mechanical disruption protocol.

Different protocols for mechanical disruption of tissues are available [38]. These methods can be roughly divided into four groups: grinding, shearing, beating, and shocking. We first tested the shearing method on a human FF pancreas and vulva carcinoma CNB as well as on two colon carcinoma CNB. Biopsies were vortexed in a cold room for 1 h. This method did not result in dissociation of the tumor tissue. Subsequently, we tried to shear the same tumor tissue with a needle and syringe; however, this did not result in disruption of the tumor biopsy either. Even with a mortar and pestle (grinding method), we did not succeed in homogenizing the tumor tissue. These observations, together with limitations such as loss of material and potential contamination when using these methods, prompted us to evaluate other homogenization methods (procedures listed in Supplementary Materials S3). We used the TissueLyser II instrument (beating method) to disrupt BC tumor tissue. This instrument disrupts difficult-to-lyse tissues through high-speed shaking of the plastic tube containing the tumor tissue as well as RNase-free stainless-steel beads. Additional advantages of this technology are that more samples can be disrupted at once, thus enhancing throughput. Furthermore, the method should be highly reproducible as it is automated and compatible with downstream RNA purification protocols. Sometimes obtaining the desired degree of homogenization required the additional use of a rotor-stator called the TissueRuptor II instrument (shearing method). Next, we extracted RNA using the Qiagen RNeasy mini kit as recommended in the nanoString nCounter guidelines. The Qubit fluorometer was used to measure the level of RNA, as the dyes used in this assay allow accurate assessment of RNA yields after excitation using the red channel [39].

**Figure 3:**
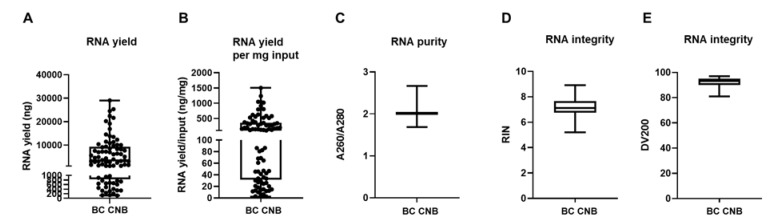
Mechanical disruption of fresh-frozen core needle BC samples allows RNA extraction. (**A**) Graph summarizing variation of RNA yield expressed in ng by depicting the minimum value, first quartile (Q1), median, third quartile (Q3), and maximum value of RNA yield. Each sample for which RNA extraction was successful is depicted as a separate symbol. (**B**) Graph summarizing the variation of RNA yield per mg input by depicting the minimum value, first quartile (Q1), median, third quartile (Q3), and maximum value of RNA yield per mg input material (ng/mg). (**C**) Graph summarizing RNA purity by depicting the minimum value, Q1, median, Q3, and maximum value of A260/A280. (**D**,**E**) Graph summarizing the integrity of the obtained RNA by depicting the minimum value, Q1, median, Q3, and maximum of the RIN and DV200 values, respectively (*n* = 81).

We extracted RNA in 81 out of 88 samples (success rate of 92%) with a yield of 5572 ng on average (range of 126 to 29,040 ng; depicted as minimum and maximum value in Figure 3A). When we normalize for input material (weight of CNB), we obtain a yield of 256 ng/mg input material on average (range of 2.41 to 1501 ng/mg; depicted as minimum and maximum value in Figure 3B). The obtained success rate is similar to an earlier provided RNA extraction protocol from BC CNB, but RNA output levels exceeded the reported concentrations, which might be attributed to the progression made in, e.g., homogenization methods over the years [40]. The A260/A280 ratio of these RNA samples was 2.012 on average (range of 1.688 to 2.667, Figure 3C). Based on this parameter, the RNA was considered to be of good quality [41]. Further analysis of the RNA using the Agilent 2100 Bioanalyzer RNA 6000 Nano LabChip showed that 92.6% of the samples had a RIN value equal or higher than 6 (on average 7.15, range of 5.2 to 8.9; Figure 3D). These RIN values signified that the RNA could be used for GEP [42]. This was corroborated by the DV200 values, which were consistently above 80% (on average 92.37%, range of 81 to 97%; Figure 3E), demonstrating that the RNA was of high quality and trustworthy for GEP analysis [43].

### 3.2. The Tissue Composition of Snap Frozen Core Needle Biopsies Affects RNA Yield

**Figure 4:**
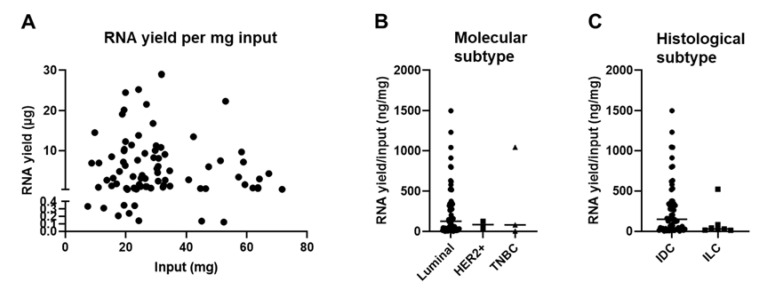
Tissue composition of snap-frozen core needle biopsies affects RNA yield. (**A**) Graph summarizing the RNA yield (y-axis, µg) in function of the amount of input material (x-axis, mg) for all samples for which RNA was obtained (*n* = 81). The nonparametric Spearman correlation was calculated for samples for which RNA extraction was successful to evaluate the relationships between yield and input. The Spearman R was −0.048 with a two-tailed *p*-value of 0.617. (**B**) Graph summarizing the RNA yield per input (ng/mg) for all samples divided in the BC molecular subtypes; luminal A + B (*n* = 65), HER2^+^ (*n* = 2), and TNBC (*n* = 3). The Kruskal–Wallis test was applied to evaluate significance and a *p*-value of 0.936 was obtained. Each symbol represents an individual sample. (**C**) Graph summarizing the RNA yield per input (ng/mg) for all samples divided in the BC histological subtypes; invasive ductal BC (*n* = 60) and invasive lobular BC (*n* = 7). The Mann–Whitney test was performed to evaluate significance and a *p*-value of 0.062 was obtained. Each symbol represents an individual sample.

We did not find a positive correlation between the amount of extracted RNA and the weight of the FF CNB (Figure 4A), which is in contrast to other reports in which it was shown that the size of the biopsy positively correlated with the RNA yield, at least for samples of the skin, adipose tissue, liver, and lung tumors [44,45,46]. We hypothesized that this variability could be linked to the heterogeneity of the BC tumor samples used in this study. These are variable in terms of molecular subtype; therefore, the composition of the tumor microenvironment (TME) differs considerably [47,48,49,50,51]. The TME can be considered a heterogeneous ecosystem composed of infiltrating immune cells, mesenchymal support cells, and matrix components. In addition, adipocytes are often abundantly present in BC samples [47,52,53]. The tissue type can thus significantly influence the overall cellularity of a sample, which can be directly correlated with yield. To gain insight on this issue, we set out to compare the RNA yield per mg input tissue between samples of different molecular BC types. The sample number for the HER2^+^ (*n* = 2) and TNBC (*n* = 3) subtype was small in comparison to the luminal A and B subtypes (*n* = 65). Therefore, it is prudent not to draw strong conclusions, although we provide Figure 4B to show the results. As an alternative, we evaluated the RNA yield per mg input tissue between samples categorized as invasive ductal BC (IDC) (*n* = 60) or invasive lobular BC (ILC) (*n* = 7) based on histology. Only one patient was referential for invasive papillar BC, invasive mucinous BC, and intraductal papilloma histological subtype (in total *n* = 3) and therefore not included in the graph. Figure 4C shows that the RNA yield per mg input tissue was on average higher in samples categorized as IDC (on average 276.3, range of 2.41 to 1501 ng/mg input) when compared to ILC (on average 103.4, range of 13.05 to 523.3 µg/mg input). The *p*-value of 0.0618 suggested a potential trend that the tissue composition could affect the RNA yield.

### 3.3. FFPE Samples Can Serve as a Back-Up Source for RNA Extraction

In 8% of cases the extracted RNA from FF BC CNB was insufficient in yield, purity, and/or integrity. In a clinical setting, FFPE specimens are generated routinely for histopathology purposes and entail large amounts of clinically relevant data. Therefore, we evaluated whether sufficient RNA could be extracted from FFPE samples when RNA extraction from FF BC samples fails.

**Figure 5:**
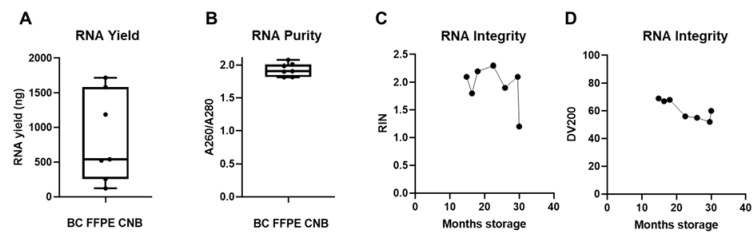
FFPE samples can serve as back-up starting material when RNA extraction from snap-frozen core needle biopsies fails. (**A**) Graph summarizing the RNA yield by depicting the minimum value, Q1, median, Q3, and maximum value of RNA yield from patients in which RNA extraction from FF BC CNB failed (*n* = 7). Each symbol represents an individual sample. (**B**) Graph summarizing RNA purity by depicting the minimum value, Q1, median, Q3, and maximum value of A260/A280. (**C**,**D**) Graph summarizing the integrity of the obtained RNA (*n* = 7) by depicting the RIN and DV200 values in function of archiving time (represented on the x-axis as months of storage at room temperature (RT)).

To further guarantee the quality of the RNA extracted from FFPE specimens, it was of utmost importance to avoid ribonuclease (RNase) contamination during the RNA isolation process. We first optimized and validated a protocol for microtome sectioning in an RNase-free manner (Supplementary Methods 2). In function of the downstream nanoString nCounter^®^ assay, we consulted input parameter recommendations. Since a maximum input of 40 µm slides is suggested, we used two sections of 20 µm thickness as input, ensuring a higher percentage of intact cells in larger sections and efficient deparaffinization, and subsequently followed the Qiagen RNeasy FFPE kit specifications for RNA extraction. We extracted sufficient amounts of RNA (success rate of 100%) with a yield of, on average, 848.13 ng (range of 122.32 to 1720.4 ng) to use in downstream nanoString nCounter^®^ GEP analysis for all seven samples (Figure 5A), but the RNA yield was remarkably lower than that derived from FF CNB (Figure 3A). The A260/A280 ratio of these RNA samples was on average 1.929 (range of 1.816 to 2.073), indicating pure RNA output (Figure 5B). Further quality analysis concerning RNA integrity showed an average RIN value of 1.94 (range of 1.2 to 2.3) and an average DV200 value of 61 (range of 52 to 69) (Figure 5C,D). This confirms that RNA extraction from FFPE samples resulted in higher RNA fragmentation than RNA extraction from FF CNB, as reflected in the lower RIN and DV200 values (Figure 5C,D) compared to the RIN values derived from FF core needle BC samples (Figure 1D,E). These quality parameters are in line with previous reports. Abramovitz et al. compared different commercially available RNA extraction kits, and using the Qiagen RNeasy FFPE kit with proteinase K step for 15 min, RNA was obtained with an average A260/A280 value of 1.9 (1.7–2.0) and RIN value of 1.7 (0–2.4) [54]. Importantly, a negative correlation between archiving time and RNA quality (RIN values) was observed, confirming earlier findings that tissue fixation time in addition to specimen size and tissue storage conditions are important factors influencing RNA quality [27,30]. Figure 3C shows a decrease in RIN values when FFPE specimen storage time at room temperature (RT) reached 30 months. RNA fragment length showed higher degradation kinetics when 20 months of RT storage was exceeded, as evidenced by the gradual decline in DV200 value (Figure 5D). Notably, nanoString nCounter^®^ technology was demonstrated to also work well with old FFPE tissue-derived RNA [55]. In addition, the obtained RNA quality parameters answered the requirements to perform nanoString nCounter^®^ GEP (A260/A280 ratio ~1.8 for RNA and DV200 ≥ 50; described in MAN-10050-03).

### 3.4. RNA Extracted from FF or FFPE BC Core Needle Biopsies Can Be Used in nanoString nCounter^®^ Technology

Most studies focus on the use of clinically available FFPE specimens as starting material to perform downstream GEP [31]. Exceptionally, FF CNB are available and used for molecular profiling. Therefore, we evaluated the feasibility of both sample types to be used in nanoString nCounter^®^ technology. Due to the various efforts made in the field of molecular biology, required input levels have remarkably decreased; only 50 ng of RNA input (derived from FF CNB) is required to perform a nanoString nCounter^®^ assay, which is a tenfold decrease compared to the microarray analysis described in 2002 by Ellis et al. [40]. This allows different potential read-outs to be combined and molecular profiling of the tumor specimen to be expanded.

**Figure 6:**
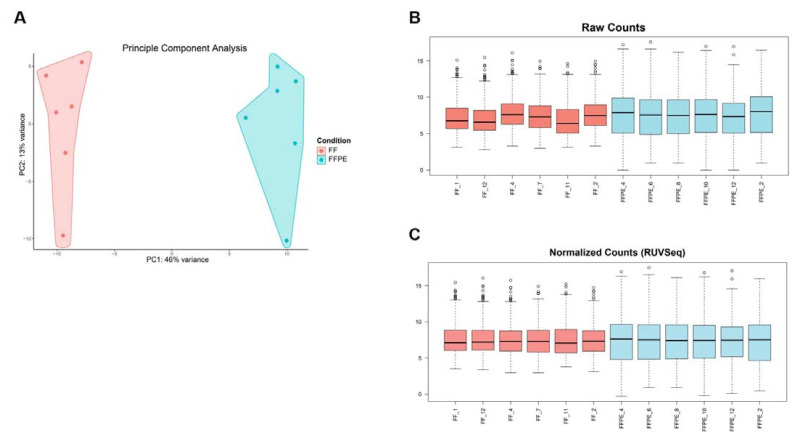
RNA derived from FF or FFPE BC core needle biopsies can be used in the nanoString nCounter^®^ technology; however, they are best not combined for comparison of GEP. (**A**) Graph representing principal component analysis (PCA) with principle component (PC)1 on the x-axis indicating 46% variance and PC2 on the y-axis indicating 13% of variance. Independent BC samples are colored by sample type (FF samples in pink, FFPE samples in blue, *n* = 12). (**B**) Boxplots depicting variation of raw counts of FF (*n* = 6) and FFPE (*n* = 6) samples of all included genes (*n* = 770). The median value and upper and lower quartiles are represented. Whiskers were drawn according to the Tukey method. (**C**) Boxplots depicting normalized counts of FF (*n* = 6) and FFPE (*n* = 6) samples using the RUV methodology (RUVSeq package).

To ensure correct GEP analysis, we first assessed different characteristics (concentration, purity, RNA integrity, size distribution of RNA fragments, DV200 values) of the extracted RNA from FF as well as FFPE samples and integrated this as standard control prior to GEP. Since formalin fixation introduces crosslinks between molecules and modification of RNA by formaldehyde, which can impair downstream assays, or the presence of gDNA contamination can affect the accuracy of GEP, it is important to evaluate RNA expression to predict cellular biological functions of the extracted RNA [27]. Therefore, we verified the gene signal variation of the extracted RNA originating from different FF and FFPE BC CNB (*n* = 12) by analyzing expression of 770 genes included in the Human PanCancer Immune Profiling panel using nanoString nCounter^®^ technology. The results confirmed that RNA derived from both sample types is suited for downstream nanoString nCounter^®^ GEP analysis [37,56]. We observed that sample types clustered together, indicating variation between sample types (Figure 6A). It has already been acknowledged that formalin fixation is associated with transcriptome changes, but expression measurements were altered in a generally consistent manner by formalin fixation across tissue types, referring to the “box length” or “intra-patient” variability depicted in Figure 6B [32]. To counteract (technical) variability, raw counts were normalized, making use of 40 housekeeping genes included in the Human PanCancer Immune Profiling Panel, and the remove unwanted variation (RUV) methodology was applied (Figure 6C) [37]. Samples were now validated for downstream analyses, but the formalin-fixation effect (box length) for FFPE derived samples was still present. Nonetheless, we concluded that RNA from both sample types can be used for downstream nanoString nCounter^®^ GEP. Our conclusion is consistent with Wimmer et al., who saw highly reproducible and concordant gene behavior in FF and FFPE microarrays, supporting the validity of using two sample types for GEP [30]. As the use of two sample types as a source of RNA for GEP can introduce bias, we recommend focusing on one sample type. If both sample types are incorporated, comparative analysis needs to be carefully and consistently performed. Specimens should be limited to those derived from a similar (preservation as well as processing) protocol and input levels for FFPE-derived samples should be corrected. In addition, the integration of data for combined analyses across FFPE/FF and platforms is feasible when batch correction methods are applied [55]. Notably, it is important when sample types are combined to ensure that clustering occurs due to biological effects and not due to the fixation method.

## 4. Conclusions

Most studies focus on the use of clinically available FFPE specimens for downstream multigene analysis. Exceptionally, FF CNB are available. However, comprehensive protocols with a high level of detail that are easy to use and allow an unexperienced researcher to successfully extract RNA from precious clinical samples are not yet available. Therefore, we provided a standardized approach for FF as well as FFPE core needle sample processing and downstream RNA extraction compatible with nanoString nCounter^®^ GEP. This optimized RNA extraction protocol resulted, in the case of FF BC CNB, in 92% successful RNA extractions. For the remaining 8%, FFPE specimens served as a back-up plan; we were also able to extract sufficient RNA to perform downstream GEP.

Different parameters such as (1) starting material, (2) sample handling, (3) requirements of downstream assays, and (4) lab equipment determine the choice of RNA extraction method. We observed that the critical parameter for successful RNA extraction starting from FF CNB is complete dissociation of the tumor CNB. For an RNA extraction procedure starting from FFPE specimens, RNase contamination is a major wrongdoer. More specifically, FFPE specimens are more prone to fragmentation, and therefore it is highly recommended to counteract further degradation of the RNA by macrodissecting FFPE specimens in an RNase-free manner. Therefore, we provided standardized operating procedures to optimally extract RNA starting from FF or FFPE tumor specimens. In both scenarios, we maximized RNA output from minimal input. Moreover, we validated the use of RNA derived from FF as well as FFPE for GEP by characterizing different quality parameters as functionality.

Advantages of this protocol: This protocol allows RNA to be extracted from small, difficult-to-lyse FF biopsies in 92% of cases and from FFPE-generated specimens—both compliant with downstream nanoString GEP.

Limitations of this protocol: Specific lab equipment is required because homogenization is a success-determining factor.

Time considerations of this protocol: The basic protocol concerning tissue homogenization and RNA extraction from FF CNB can be completed in two days. Homogenization, overnight lysis, and the vortex step for 1 h are the most time-consuming steps. The duration of the RNase-free macrodissection protocol is dependent on the number of samples and the number of times the procedure needs to be repeated; for one FFPE specimen, this work takes another 30 min in addition to the macrodissection itself.

Based on these results we were able to provide an experimental workflow of RNA isolation of FF and FFPE core needle BC biopsies. Both RNA derived from FF and FFPE BC CNB are valid sources to be used as starting material for downstream nanoString nCounter^®^ GEP.

## 5. Patents

K.T. holds a patent for dendritic cells electroporated with tumor antigen mRNA and TriMix (WO2009/034172). No other potential conflicts of interest were disclosed by the other authors.

## Data Availability

All data generated or analyzed during this study are included in this published article.

## Supplementary Materials

The following are available online at https://www.mdpi.com/article/10.3390/biom11050621/s1, Supplementary methods to extract RNA from FF and FFPE core needle biopsies are provided as standard operating procedures in S1 and S2, respectively and different homogenization protocols are described in Supplementary methods S3.

## Abbreviations

BC	breast cancer
βME	β-mercaptoethanol
CNB	core needle biopsy
ER	estrogen receptor
EtOH	ethanol
FFPE	formalin-fixed paraffin-embedded
FF	fresh-frozen
GEP	gene expression profiling
HER2	human epidermal growth factor receptor 2
IDC	invasive ductal carcinoma
ILC	invasive lobular carcinoma
IRG	immune-related gene
PCA	principal component analysis
rcf	relative centrifugal force
RIN	RNA integrity number
RNA	ribonucleic acid
RNase	ribonuclease
RUV	remove unwanted variation
SOP	standard operating procedure
UZ Brussel	Universitair Ziekenhuis Brussel
VUB	Vrije Universiteit Brussel

## Appendix A

**Table A1:** Nomenclature and safety measurements of the reagents used in the different protocols.

Hazardous Compound	Kit/Buffer	Storage	Danger	Precaution	Action
**2-mercaptoethanol**	Added to RLT buffer (Qiagen RNeasy kit)	2–8 °C Store in a well-ventilated place. Keep container tightly closed.	Toxic if swallowed or if inhaled. Fatal in contact with skin, causes skin reaction, may cause an allergic skin reaction. Causes serious eye damage. May cause damage to organs through prolonged or repeated exposure if swallowed. Very toxic to aquatic life, with long-lasting effects.	Avoid breathing dust/fume/gas/mist/vapors/spray. Wear protective gloves/protective clothing/eye protection/face protection.	If swallowed: Immediately call a poison center/doctor, rinse mouth. If on skin: Wash with plenty of water, immediately call a poison center/doctor. If in eyes: Rinse cautiously with water for several minutes. Remove contact lenses if present and easy to do. Continue rinsing. Immediately call a poison center/doctor.
**Sodium dodecyl sulfate**	RNase ZAP	15–25 °C	Causes mild skin irritation.	Wear protective gloves/protective clothing/eye protection/face protection.	If skin irritation occurs get medical advice/attention.
**Ethanol (100%)**	Added to buffer RPE (Qiagen RNeasy kit). Added (70% EtOH) to lysate (Qiagen RNeasy kit) 100% EtOH as such (RNase free macrodissection) Added to buffer RPE (Qiagen RNeasy FFPE kit) 100% EtOH to sample (Qiagen RNeasy FFPE kit)	15–25 °C Store in a well-ventilated place. Keep container tightly closed.	Highly flammable liquid and vapor. Causes serious eye irritation.	Keep away from heat/sparks/open flames/hot surfaces. No smoking. Ground/bond container and receiving equipment.	If in eyes: Rinse cautiously with water for several minutes. Remove contact lenses if present and easy to do. Continue rinsing.
**Guanidine thiocyanate** **Guanidine thiocyanate +** **Ethanol**	Buffer RLT (Qiagen RNeasy kit) Buffer RW (Qiagen RNeasy kit)	15–25 °C	Flammable liquid and vapor. Harmful if swallowed. May be harmful in contact with skin or if inhaled. Causes serious eye damage. Harmful to aquatic life, with long-lasting effects. Contact with acids liberates very toxic gas.	Keep away from heat/sparks/open flames/hot surfaces. No smoking. Wear protective gloves/protective clothing/eye protection/face protection.	If in eyes: Rinse cautiously with water for several minutes. Remove contact lenses, if present and easy to do. Continue rinsing. Immediately call a poison center or doctor/physician.
**Guanidium chloride**	Buffer RBC (Qiagen RNeasy FFPE kit)	Store in a well-ventilated place	Causes skin irritation. Causes serious eye irritation. Harmful if swallowed or inhaled.	Avoid breathing dust/fume/gas/mist/vapors/spray. Wear protective gloves/protective clothing/eye protection/face protection. Wear respiratory protection.	If inhaled: If breathing is difficult, remove victim to fresh air and keep at rest in a position comfortable for breathing. If eye irritation persists: Get medical advice/attention. If respiratory symptoms: Call a poison center or doctor/physician. Take off contaminated clothing and wash it before reuse. Dispose of contents/container to an approved waste disposal plant.
**DNase I**	DNase I (Qiagen RNeasy (FFPE) kit)	Store in a well-ventilated place	May cause an allergic skin reaction. May cause allergy or asthma symptoms or breathing difficulties if inhaled.	Avoid breathing dust/fume/gas/mist/vapors/spray. Wear protective gloves/protective clothing/eye protection/face protection. Wear respiratory protection.	If inhaled: If breathing is difficult, remove victim to fresh air and keep at rest in a position comfortable for breathing. If eye irritation persists: Get medical advice/attention. If respiratory symptoms: Call a poison center or doctor/physician. Take off contaminated clothing and wash it before reuse. Dispose of contents/container to an approved waste disposal plant.
**Proteinase K**	Proteinase K (Qiagen RNeasy FFPE kit)	Store in a well-ventilated place	May cause allergy or asthma symptoms or breathing difficulties if inhaled. Causes mild skin irritation.	Avoid breathing dust/fume/gas/mist/vapors/spray. Wear protective gloves/protective clothing/eye protection/face protection. Wear respiratory protection.	If inhaled: If breathing is difficult, remove victim to fresh air and keep at rest in a position comfortable for breathing. If eye irritation persists: Get medical advice/attention. If respiratory symptoms: Call a poison center or doctor/physician. Take off contaminated clothing and wash it before reuse. Dispose of contents/container to an approved waste disposal plant.
**Dimethyl sulfoxide (DMSO)**	Qubit RNA HS assay kit (RNA reagent, 20× concentrated) Agilent RNA 6000 nano/pico kit (dye/gel)	Store in a well-ventilated place.	Combustible liquid	Keep away from heat/hot surfaces/sparks/open flames and other ignition sources. No smoking. Wear protective gloves/protective clothing/eye protection/face protection.	In case of fire: Use dry sand, dry chemical, or alcohol-resistant foam for extinction. Dispose of contents/container to an approved waste disposal plant.
**Sodium hydroxide**	NaOH as such (RNase free macrodissection)	Store in a cool, dry, well-ventilated place. Separate from incompatible materials. Keep container tightly closed.	Highly reactive. Incompatible with many common chemicals. Reacts violently with water. Contact with metals liberates flammable hydrogen gas. Extremely corrosive. Causes severe skin burns and eye damage (resulting in blindness). If airborne, dust or mist can cause severe irritation of the nose and throat. If ingested, can burn lips, tongue, throat, and stomach and be fatal.	Avoid direct skin contact and wear protective clothing. Avoid direct contact. Wear chemical protective gloves/eye protection.	If on skin: Take off contaminated clothing, shoes quickly and gently blot or brush away excess chemical. Immediately flush with lukewarm, gently flowing water for at least 60 min. Do not interrupt flushing. If it can be done safely, continue flushing during transport to hospital. Immediately call a poison center or doctor. If in eyes: Quickly and gently blot or brush chemical off the face. Immediately flush the contaminated eye(s) with lukewarm, gently flowing water for at least 60 min while holding the eyelid(s) open. If a contact lens is present do not delay flushing or attempt to remove the lens. Take care not to rinse contaminated water into the unaffected eye or onto the face. Immediately call a poison center or doctor. If in eyes: Quickly and gently blot or brush chemical off the face. Immediately flush the contaminated eye(s) with lukewarm, gently flowing water for at least 60 min while holding the eyelid(s) open. If a contact lens is present do not delay flushing or attempt to remove the lens. Take care not to rinse contaminated water into the unaffected eye or onto the face. Immediately call a poison center or doctor. If ingested: Rinse mouth with water. If vomiting occurs naturally, have victim lean forward to reduce risk of aspiration. Have victim rinse mouth with water again. Immediately call a poison center or doctor.
**Hexadecane**	Deparaffinization solution	Store in a dry and well-ventilated place. Keep container tightly closed.	May be harmful if inhaled. Repeatedly exposure can cause dry or burst skin Can be fatal when compound ends up in airways when swallowing.	Avoid breathing dust/fume/gas/mist/vapors/spray. Wear protective gloves/protective clothing/eye protection/face protection.	If swallowed: Contact a poison center or doctor/physician immediately, rinse mouth with water. If on skin: Wash with soap and an excess of water, remove all contaminated clothing and shoes. If symptoms are persistent, contact a doctor/physician. If in eyes: Remove contact lenses if present and easy to do. Rinse thoroughly with an excess of water for at least 15 min and contact a doctor/physician.
