# Supplementary material for: Overcoming the Challenges of High Quality RNA Extraction from Core Needle Biopsy
Source: Biomolecules. 2021 Apr 22;11(5):621. doi: 10.3390/biom11050621 (PMC8143498; doi:10.3390/biom11050621)
Supplement: Supplementary file 1 [file biomolecules-11-00621-s001.zip › Supplementary Materials/Supplementary material S2_SOP_RNA extraction from FFPE core needle biopsies.pdf]

RNA extraction from FFPE core needle biopsies

LMCT LABORATORY FOR MOLECULAR & CELLULAR THERAPY RESEARCH GROUP

Role	Name and function	Date	Signature
Author	Hanne Locy, PhD student<br>Laboratory for Molecular and Cellular Therapy	22/12/2020	DocuSigned by: Hanne Locy 917193926EE6448...
The author's approval confirms that the content of this document is complete, accurate and correct from the technical point of view.			
Reviewer 1	Karine Breckpot; Laboratory director<br>Laboratory for Molecular and Cellular Therapy	23/12/2020	DocuSigned by: Karine Breckpot 0E99BCB2094F4E9...
The approval by the laboratory director confirms that this document has been reviewed and complies with good documentation practices and complies with applicable regulations.			
Reviewer 2	Rohann J.M. Correa; Radiation Oncology<br>Resident (London Health Sciences Centre)	28/01/2021	[signature]
The approval by the radiation oncology resident confirms that this document has been reviewed and complies with good documentation practices and complies with applicable regulations.			

Content of the document

1. Document History 1
2. Scope of the standard operating procedure (SOP) 1
3. Responsibilities 1
4. Purpose of the procedure 1
5. Abbreviations and definitions 1
6. Related documents 2
7. Materials 2
8. Guidelines 3
9. Procedure 3
   9.1. Solution preparation 3
   9.2. RNase free preparation of instruments
   9.3 RNase free macrodissection
   9.4 Total RNA extraction using RNeasy FFPE kit
10. Annex 4

DocuSign Envelope ID: 3C3D58C7-26F3-49AD-9866-6324E484D644

RNA extraction from FFPE core needle biopsies

1. Document History

Version	Effective Date	Changes
1	22/12/2020	First version

2. Scope of the standard operating procedure (SOP)

This SOP is part of the Quality Management System of the Laboratory for Molecular and Cellular Therapy. The procedure described in this document can only be performed by personnel trained in good clinical practice (GCP).

3. Responsibilities

Function	Responsibility
Author	Writing, conservation, update and distribution of this document.
Reviewer	Review and approval of this document.
Executor	Execution of the procedure.

4. Purpose of the procedure

This procedure describes ribonuclease (RNase) free macrodissection of Formalin-Fixed Paraffin-Embedded (FFPE) core needle biopsies (CNB) and total RNA extraction from FFPE core needle specimens using the RNeasy FFPE kit from Qiagen.

5. Abbreviations and definitions

CNB	Core needle biopsy
FFPE	Formalin-Fixed Paraffin-Embedded
i.t.	Intratumoral
M	Molar
mL	Milliliter
mRNA	Messenger ribonucleic acid
RNA	Ribonucleic acid
RNase	Ribonuclease
µm	Micrometer

6. Related documents

Document title	Description
Clinical study protocol	This document describes the trial protocol of the phase I trial entitled "A Phase I study on the safety and immune-modulatory effect of intratumoral (i.t.) administration of mRNA encoding dendritic cell activating proteins in patients with early, resectable breast cancer" in which the core needle biopsy sampling is described.
Unlocking your FFPE archive (Qiagen)	This document describes critical factors for molecular analysis of FFPE samples
RNeasy FFPE Handbook (Qiagen)	This document describes all parameters involved in total RNA extraction process from FFPE tissue sections
Working with RNA: the basics (technical note by Invitrogen)	This document describes the basics to avoid, detect and inhibit RNases.

7. Materials

**Equipment**	**Step**
Horizontal laminar flow cabinet (Esco Global)	9.1
Microtome (Thermo Scientific, HM450)	9.2
Microcentrifuge (Eppendorf, 5418R)	9.4
Spin centrifuge (Fisher Scientific)	9.4
Vortex mixer (Cleaver Scientific, CSLVORTEX)	9.4
Thermal heater (Eppendorf Thermostat plus)	9.4
Analytical scale (Sartorius, CP124S)	9.1.1
Calibrated adjustable precision pipettes dedicated pipettes for RNA work (and tips)(10, 200 and 1000 µl)	9.4
1mL or 2mL Eppendorf tube rack	9.3/9.4
**Materials**	**Step**
Ice	9.4
Gloves	9
Forceps	9.2/9.3
Disposable blades (Thermo Scientific, 152200)	9.2.9
Baked (at 232°C) Pyrex glass bottles	9.1/9.2
Filter tips (Neptune Scientific, BT1000.96, BT200, BT10XL)	9.4
1.5mL Eppendorf DNA LoBind tube (Sigma-Aldrich, EP0030108051)	9.3/9.4
2mL Safe-Lock Eppendorf tube (Eppendorf, 0030121686)	9.4
Kimberly-Clark KimWipe disposable tissue (Merck, Z188956)	9.2/9.3
**Reagents**	**Step**
RNase ZAP spray (Invitrogen, AM9782)	9.2/9.3/9.4
Qiagen RNeasy FFPE kit (Qiagen, 73504)	9.4

DocuSign Envelope ID: 3C3D58C7-26F3-49AD-9866-6324E484D644

Deparaffinization clean lab solution (VWR, 10047400)	9.2.2
NaOH (Sigma-Aldrich, 06203)	9.1/9.2
MilliQ water	9.1/9.2
100% Ethanol	9.1/9.2/9.4

8. Guidelines

- RNase free working and RNase free work area (as described in technical note of Invitrogen 'Working with RNA: the basics') – some highlights:
  - Dedicate a set of pipettes solely used for RNA work; use RNase-free tips, tubes, chemicals and reagents; work in a 'designated RNase-free zone' defined as a low-traffic area, away or shielded from air vents or open windows
  - Avoid sources of RNase contamination, such as bodily fluids (e.g., skin oils) by gloving your hands, decontaminating gloves using RNase ZAP wipes and frequently changing, and by wearing a laboratory coat
  - Keeping in mind the robust nature of RNases, glassware and metalware need to be treated with RNase ZAP reagent or need to be baked at 232.2°C for 2 hours or more.
- Different considerations should be taken when preparing, archiving and retrieving usable analytes from FFPE specimens. Here summarized in a table based on 'Unlocking your FFPE archive' document from Qiagen:

**Preparation and archiving FFPE samples**		
**FFPE workflow**	**Problem**	**Solution**
1. Sample type	Different sample (tissue) types (fibrous, fatty,…). In case tumorous tissue: ratio (non)-malignant cells heterogenously distributed	
2. Sample handling	Degradation, induction of modification of biomolecules in the time frame between tissue removal and fixation	Duration of procedure prior to tissue fixation as short as possible
3. Fixation	Different compositions of formalin solutions inducing crosslinks between biomolec	Use of neutral-buffered formalin solution (circumventing the nucleic acid quality impairing precence degradation products of unbuffered or acidic formalin solutions)
	Underfixation of tissue leading to nucleic acid and protein degradation or changes in gene expression, overfixation leading to more extensive crosslinking (hampering extraction of usable nucleic acids and proteins)	Optimal conditions; maximum 5mm thick tissue specimen, ratio formalin: tissue (10:1), maximum 24 hours fixation, adaption of formalin volume
4. Embedding	Residual water present prior embedding, leading to sample degradation and proteolysis	Use of high-quality reagents undiluted with water and adapted duration and temperature of embedding process
	Different melting temperatures (Tm) of paraffins; high required Tm resulting in increased sample degradation	Use of low-melting temperature paraffin without additives
5. Storage	Storage at room temperature leading to higher fragmentation	Storage at 4°C slowing down degradation of nucleic acids and proteins

**Retrieval and analysis of analytes from FFPE samples**		
1. Deparaffinization	Different deparaffinization methods dependent on downstream purification proced	In case of DNA or RNA purification; various hydrophobic solvents can be used(xylene/heptane/limonene) or Qiangs deparaffinization solution or heating
2. Purification	Biomolecules are crosslinked to each other (extent partly dependent on duration of formalin fixation) and chemical modifications due to crosslinking	Release of RNA molecules from crosslinked protein molecules using proteinase K treatment (at 56°C, 15 minutes) and reversing chemical modifications by incubation at 80°C
3. Molecular analysis-	Purity	Ratio of absorbances at 260nm and 280nm and 260nm and 230nm using spectrophotometer
	Concentration	Concentration measurement using fluorometric method
	RNA integrity	Measurement of RIN value and DV200 value using Agilent technologies
	RNA degradation and functionality	Gene expression analysis by RT-PCR

- Avoid cross-contamination between different FFPE core needle specimens originating from different patients by renewing gloves, frequently perform RNase free treatment, use of different tips.

9. Procedure

9.1. Solution preparation

9.1.1. Prepare 100mL of 1M NaOH and transfer to a baked (at 232°C) Pyrex glass bottle

9.1.2. Transfer 100mL of MilliQ water and 100% Ethanol to baked Pyrex glass bottle

9.2. RNase free preparation of instruments

9.2.1 Remove the previous present blade

9.2.2 De-paraffinate the microtome with the deparaffinization clean lab solution

9.2.3 Change gloves and spray gloves with RNase ZAP. Allow to briefly air dry

9.2.4 Clear work area/benchtop by spraying RNase ZAP to disposable KimWipe and wipe benchtop surface in immediate work area

9.2.5 Clean surfaces of foreceps using an RNase ZAP wipe and replace foreceps back on a fresh disposable KimWipe flat placed on the lab bench

NOTE: Make sure that RNase ZAP reagent is not in contact with surfaces of forceps, spatulas or other reactive metalware (e.g. aluminium) for more than a few minutes to avoid corrosion.

9.2.6 Clean microtome with an RNase ZAP wipe; clean thoroughly the zones of the microtome that comes in contact with the slide from FFPE specimen

9.2.7 Additionally clean microtome and foreceps subsequently with:

1. A soaked KimWipe with NaOH solution
2. A soaked KimWipe with MilliQ water
3. A soaked KimWipe with 100% ethanol

9.2.8 Allow microtome to briefly air dry and place foreceps back on a dry residing KimWipe

9.2.9 Take a new blade carefully at blunt edge and treat subsequently with:

1. RNase ZAP spray. Cean excess RNase ZAP with a new KimWipe
2. A soaked KimWipe with NaOH solution
3. A soaked KimWipe with MilliQ water
4. A soaked KimWipe with 100% ethanol

9.2.10 Mount blade

9.3 RNase free macrodissection

9.3.1 Spray gloves with RNase ZAP prior to handling

9.3.2 Mount patient specific FFPE block

9.3.3 Begin initial 'trimming' of FFPE block to reach desired depth within until the total surface of tissue is exhibited

9.3.4 Once desired depth is reached, begin cutting for curls

9.3.5 Transfer 2x20µm slides to an 1.5mL Eppendorf DNA LoBind tube using RNase free foreceps and close immediately

9.4 Total RNA extraction using RNeasy FFPE kit

9.4.1 Initiate total RNA extraction from FFPE CNB by deparaffinizing

NOTES: (1) The first step of the Qiagen Quick-Start total RNA extraction from FFPE specimen protocol is the deparaffinization of the curls. Two methods, the deparaffinization solution from Qiagen and the heptane/methanol method (described in Appendix A from the RNeasy FFPE handbook), can be used to extracte total RNA. (2) To increase the RNA yield, eluate was reloaded on the elution column.

10. Annex

The Quick-Start Protocol to extract total RNA from FFPE (tumor) tissue using the RNeasy FFPE kit from Qiagen is attached in annex.

DocuSign Envelope ID: 3C3D58C7-26F3-49AD-9866-6324E484D644

February 2019

Quick-Start Protocol

RNeasy® FFPE Kit

RNase-Free DNase I and RNeasy MinElute® spin columns should be stored at 2–8°C upon arrival. All other reagents and components of the RNeasy FFPE Kit (cat. no. 73504) should be stored at room temperature (15–25°C). Proteinase K is stable for at least 1 year after delivery when stored at room temperature. If longer storage is required or if ambient temperatures often exceed 25°C, we recommend storage at 2–8°C.

Further information

- *RNeasy FFPE Handbook*: **www.qiagen.com/HB-0375**
- Safety Data Sheets: **www.qiagen.com/safety**
- Technical assistance: **support.qiagen.com**

Notes before starting

- This protocol is for the purification of total RNA from FFPE tissue sections. For purifying total RNA from microdissected FFPE tissue sections, refer to the *RNeasy FFPE Handbook*.
- Buffer RBC contains a guanidine salt and is therefore not compatible with disinfecting reagents containing bleach. See the "Safety Information" section in the *RNeasy FFPE Handbook*.
- Unless otherwise indicated, all steps should be performed at room temperature (15–25°C). Work quickly.
- Perform all centrifugation steps using a microcentrifuge set at 15–25°C. If using a refrigerated microcentrifuge, set the temperature to 20–25°C; otherwise, significant cooling below 15°C may occur.
- If using Buffer RPE and the RNase-Free DNase I for the first time, reconstitute them as described in the *RNeasy FFPE Handbook*.
- Equilibrate all buffers to room temperature (15–25°C). Mix reconstituted Buffer RPE by shaking.
- Set a thermal mixer, heat block or water bath to 56°C for use in step 5 and step 9. If possible, set a second thermal mixer, heat block or water bath to 80°C for use in step 9.
- ▲ indicates volumes to use if processing 1–2 sections per sample, while ● indicates volumes to use if processing >2 sections per sample.

1. Using a scalpel, trim excess paraffin off the sample block.
2. Cut sections 5–20 µm thick.
3. Immediately place the sections in ▲ a 1.5 ml or 2 ml microcentrifuge tube or ● a 2 ml microcentrifuge tube (not supplied) and close the lid.
4. Add ▲ 160 µl or ● 320 µl Deparaffinization Solution, vortex vigorously for 10 s and centrifuge briefly to bring the sample to the bottom of the tube.

   Note: Deparaffinization Solution is not supplied with the RNeasy FFPE Kit and should be ordered separately (cat. no. 19093).
5. Incubate at 56°C for 3 min, and then allow to cool at room temperature.
6. Add ▲ 150 µl or ● 240 µl Buffer PKD, and then mix by vortexing.
7. Centrifuge for 1 min at 11,000 x *g* (10,000 rpm).
8. Add 10 µl proteinase K to the lower, colorless phase. Mix gently by pipetting up and down.

DocuSign Envelope ID: 3C3D58C7-26F3-49AD-9866-6324E484D644

9. Incubate at 56°C for 15 min, and then at 80°C for 15 min. Ensure that the heating block has reached 80°C before starting the 15 min incubation.

   If the heating block that you used has no shaking function, vortex the mixture briefly every 3–5 min.

10. Transfer the lower, colorless phase into a new 2 ml microcentrifuge tube.
11. Incubate the mixture on ice for 3 min, and then centrifuge for 15 min at 20,000 x *g* (13,500 rpm).
12. Transfer the supernatant to a new microcentrifuge tube (not supplied). Be careful not to disturb the pellet.
13. Add DNase Booster Buffer equivalent to one-tenth of the total sample volume (approximately ▲ 16 µl or ● 25 µl) and 10 µl DNase I stock solution. Mix by inverting the tube. Centrifuge briefly to collect residual liquid from the sides of the tube.
14. Incubate at room temperature for 15 min.
15. Add ▲ 320 µl or ● 500 µl Buffer RBC to adjust binding conditions, and then mix the lysate thoroughly.
16. Add ▲ 720 µl or ● 1200 µl ethanol (100%) to the sample, and then mix well by pipetting. Do not centrifuge. Proceed immediately to step 17.
17. Transfer 700 µl of the sample – including any precipitate that may have formed – to an RNeasy MinElute spin column placed inside a 2 ml collection tube (supplied). Close the lid gently, and then centrifuge for 15 s at ≥8000 x *g* (≥10,000 rpm). Discard the flow-through. Reuse the collection tube in step 18.
18. Repeat step 17 until the entire sample has passed through the RNeasy MinElute spin column. Reuse the collection tube in step 19.
19. Add 500 µl Buffer RPE to the RNeasy MinElute spin column. Close the lid gently, and then centrifuge for 15 s at ≥8000 x *g* (≥10,000 rpm). Discard the flow-through. Reuse the collection tube in step 20.
20. Add 500 µl Buffer RPE to the RNeasy MinElute spin column. Close the lid gently, and then centrifuge for 2 min at ≥8000 x *g* (≥10,000 rpm) to wash the spin column membrane. Discard the collection tube with the flow-through.
21. Place the RNeasy MinElute spin column in a new 2 ml collection tube (supplied). Open the lid of the spin column, and centrifuge at full speed for 5 min. Discard the collection tube with the flow-through.
22. Place the RNeasy MinElute spin column in a new 1.5 ml collection tube (supplied). Add 14–30 µl RNase-free water directly to the spin column membrane. Close the lid gently, and centrifuge for 1 min at full speed to elute the RNA.

Scan QR code for handbook.

For up-to-date licensing information and product-specific disclaimers, see the respective QIAGEN kit handbook or user manual.

Trademarks: QIAGEN®, Sample to Insight®, MinElute®, RNeasy® (QIAGEN Group). Registered names, trademarks, etc. used in this document, even when not specifically marked as such, are not to be considered unprotected by law.

1116192 02/2019 HB-2634-001 © 2019 QIAGEN, all rights reserved.
