# Supplementary material for: Overcoming the Challenges of High Quality RNA Extraction from Core Needle Biopsy
Source: Biomolecules. 2021 Apr 22;11(5):621. doi: 10.3390/biom11050621 (PMC8143498; doi:10.3390/biom11050621)
Supplement: Supplementary file 1 [file biomolecules-11-00621-s001.zip › Supplementary Materials/Supplementary Materials_S3.pdf]

Supplementary Materials

Supplementary Materials and Methods – S1

1. Tissue disruption through chemical lysis
Fresh core needle biopsies (CNB) from human colon, breast and kidney tumors were transferred from the 50mL collection tube containing refrigerated 5mL RNA*later*™ to a 1.5mL Safe-Lock Eppendorf tube and were immersed in 175 µL of RNA lysis buffer (SV total RNA isolation kit, Promega) and stored for 24 hours at 4°C to allow chemical lysis. RNA was extracted immediately after chemical lysis.

2. Tissue disruption through chemical lysis and shearing of the sample
Fresh CNB from pancreas, vulva and two colon tumors were transferred from the 50mL collection tube containing refrigerated 5mL RNA*later*™ to a 1.5mL Safe-Lock Eppendorf tube and were immersed in 350 µL of RNA lysis buffer (RNeasy kit, Qiagen). Subsequently, these CNB were homogenized using a vortex (1 hour, 4°C) followed by shearing using a syringe and a 18-gauge needle. Shearing was achieved by passing the lysate 5 to 10-times through the needle. RNA was extracted immediately after this procedure.

3. Tissue disruption through grinding of the sample
Fresh CNB from two colon tumors were transferred from the 50mL collection tube containing refrigerated 5mL RNA*later*™ to a 1.5mL Safe-Lock Eppendorf tube and were snap frozen in liquid nitrogen and subsequently ground using a micro pestle (UN-81441, CP Lab Safety) for further disruption through grinding of the sample. Lysis buffer (350µL, RNeasy kit, Qiagen) was added to the homogenized sample and RNA was immediately extracted.
