# Supplementary material for: Overcoming the Challenges of High Quality RNA Extraction from Core Needle Biopsy
Source: Biomolecules. 2021 Apr 22;11(5):621. doi: 10.3390/biom11050621 (PMC8143498; doi:10.3390/biom11050621)
Supplement: Supplementary file 1 [file biomolecules-11-00621-s001.zip › Supplementary Materials/Supplementary_material_S1_SOP_RNA extraction from FF core needle biopsies.pdf]

RNA extraction from FF core needle biopsies

LMCT LABORATORY FOR MOLECULAR & CELLULAR THERAPY RESEARCH GROUP

Role	Name and function	Date	Signature
Author	Hanne Locy; PhD student<br>Laboratory for Molecular and Cellular Therapy	19/04/2021	DocuSigned by: Hanne Locy 917193926EE6448...
The author's approval confirms that the content of this document is complete, accurate and correct from the technical point of view.			
Reviewer 1	Karine Breckpot; Laboratory director<br>Laboratory for Molecular and Cellular Therapy	19/04/2021	DocuSigned by: Karine Breckpot 0E99BCB2094F4E9...
The approval by the laboratory director confirms that this document has been reviewed and complies with good documentation practices and complies with applicable regulations.			
Reviewer 2	Rohann J.M. Correa; Radiation Oncology Resident<br>London Health Sciences Centre	19/04/2021	[signature]
The approval by the radiation oncology resident confirms that this document has been reviewed and complies with good documentation practices and complies with applicable regulations.			

Content of the document

1. Document History 1
2. Scope of the standard operating procedure (SOP) 1
3. Responsibilities 1
4. Purpose of the procedure 1
5. Abbreviations and definitions 1
6. Related documents 2
7. Materials 2
8. Guidelines 3
9. Procedure 3
   - 9.1. Preparation of fresh frozen (FF) core needle biopsy
     - 9.1.1. Ultrasound guidance of core needle biopsy
     - 9.1.2. Snap freezing of core needle biopsy
   - 9.2. Homogenization and lysis of FF core needle biopsy
     - 9.2.1 Mechanical homogenization of FF core needle biopsy using TissueLyser II instrument
     - 9.2.2. Lysis of FF core needle biopsy using RLT buffer
     - 9.2.3 Additional homogenization of not fully disrupted FF core needle biopsy using TissueRuptor II dissociator
     - 9.2.4 Total RNA extraction from lysate
10. Annex 5

RNA extraction from FF core needle biopsies

1. Document History

Version	Effective Date	Changes
1	22/12/2020	First version

2. Scope of the standard operating procedure (SOP)

This SOP is part of the Quality Management System of the Laboratory for Molecular and Cellular Therapy. The procedure described in this document can only be performed by personnel trained in good clinical practice (GCP).

3. Responsibilities

Function	Responsibility
Author	Writing, conservation, update and distribution of this document.
Reviewer	Review and approval of this document.
Executor	Execution of the procedure.

4. Purpose of the procedure

This procedure describes preparatory steps, homogenization and lysis of fresh-frozen (FF) core needle biopsies (CNB) to subsequent extract total RNA using the Qiagen RNeasy kit.

5. Abbreviations and definitions

βME	β-mercaptoethanol
cm	Centimeter
CNB	Core needle biopsy
FF	Fresh-frozen
i.t.	Intratumoral
mL	Milliliter
mm	Millimeter
mRNA	Messenger ribonucleic acid
ON	Overnight
rcf	Relative centrifugal force
RLT buffer	RNA lysis buffer

RNA	Ribonucleic acid
RNase	Ribonuclease
µL	Microliter

6. Related documents

Document title	Description
Clinical study protocol	This document describes the trial protocol of the phase I trial entitled "A Phase I study on the safety and immune-modulatory effect of intratumoral (i.t.) administration of mRNA encoding dendritic cell activating proteins in patients with early, resectable breast cancer" in which the core needle biopsy sampling is described.
Working with RNA: the basics (technical note by Invitrogen)	This document describes the basics to avoid, detect and inhibit RNases.
RNeasy mini handbook	This document describes the total RNA extraction procedure making use of the Qiagen RNeasy kit.

7. Materials

Equipment	Step
Horizontal laminar flow cabinet (Esco Global)	9
Analytical scale (Sartorius, CP124S)	9.1.2 (2/6)
Centrifuge (Eppendorf 5810R)	9.1.1(3)/(9.2.3(3)
Microcentrifuge (Eppendorf, 5418R)	9.2.4
Spin centrifuge (Fisher Scientific)	9.2.1 (3)/9.2.4
Refrigerator	9.1.1(4)/9.2.2(2)
-80°C freezer	9.1.2(NOTE)
Liquid nitrogen (tank)	9.1.2(7)
Tissuelyser II instrument and adapters (Qiagen, 85300,11993)	9.2.1(2/3)
Vortex mixer (Cleaver Scientific, CSLVORTEX)	9.2.2(3)/9.2.4
Tissueruptor II dissociator (Qiagen 9002756)	9.2.3(1)
Calibrated adjustable precision dedicated pipettes for RNA work (10, 200 and 1000 µL)	9
50mL Eppendorf tube rack	9.1.1/9.2.3
1mL or 2mL Eppendorf tube rack	9.1.2/9.2.1/9.2.2/9 .2.4
**Materials**	**Step**
Disposable gloves	9
Ice	9.1.2
Filter tips (Neptune Scientific, BT1000.96, BT200, BT10XL)	9
Kimberly-Clark KimWipe disposable tissue (Merck, Z188956)	9.1.2(3)

RNase ZAP decontamination wipes (Invitrogen, AM9786)	9.1.2
RNase free disposable forceps (Heinz Merenz Medizinalbedarf Gmbh, 1131884)	9.1.2(3/6)/9.2.1(3)
Disposable scalpel (Swann-Morton, 0511)	9.1.2(5)
Sterile petri-dish (Falcon, 351029)	9.1.2 (4/5)
1.5mL Eppendorf DNA LoBind tube (Sigma-Aldrich, EP0030108051)	9.2.4
2mL Safe-Lock Eppendorf tube (Eppendorf, 0030121686)	9.2.1(1)
50mL centrifuge tube (Sarstedt, 62.547.254)	9.1.1(1)/9.2.3(1)
5mm stainless steel bead (Qiagen, 69989)	9.2.1(1)
Single-bead dispenser (Qiagen, 69965)	9.2.1(1)
Tissueruptor II probes (Qiagen, 990890)	9.2.3(1)
**Reagents**	**Location/Storage**
RNA*later*™ (Sigma-Aldrich, R0901)	9.1.1(1)
RNeasy kit (Qiagen, 74104)	9.2.2(1)/9.2.3(2)/9.2.4(1)
B-mercaptoethanol (Sigma-Aldrich, M6250)	9.2.2
96-100% Ethanol	9.2.4
70% Ethanol	9.2.4

8. Guidelines

- RNase free working and RNase free work area (as described in technical note of Invitrogen 'Working with RNA: the basics') :
  - Dedicate a set of pipettes solely used for RNA work; use RNase-free tips, tubes, chemicals and reagents; work in a 'designated RNase-free zone' defined as a low-traffic area, away or shielded from air vents or open windows
  - Avoid sources of RNase contamination, such as bodily fluids (e.g., skin oils) by gloving your hands, decontaminating gloves using RNase ZAP wipes and frequently changing, and by wearing a laboratory coat
- Store RNA*later*™ reagent at 4°C. Once biopsy is submerged in RNA*later*™ reagent, tumor biopsy can be stored for 1 day at 37°C, 1 week at 25°C, 1 month at 4°C and long-term at -20°C.
- Precool (at -80°C) the adapters of the Tissuelyser II instrument
- Avoid cross-contamination between different CNB originating from different patients by using every time different tips, disposable forceps, disposable scalpels, sterile Tissueruptor II probes,…

9. Procedure

9.1. Preparation of fresh frozen (FF) core needle biopsy (CNB)

9.1.1. Ultrasound guidance of CNB

1. Prepare in advance 50mL centrifuge tubes with 5mL RNA*later*™ solution for the radiologists
2. Once the ultrasound-guided CNB is obtained, transfer to the 50mL centrifuge tube
3. Centrifuge the 50mL tube containing CNB briefly at 375 relative centrifugal force (rcf)

DocuSign Envelope ID: 1986343D-604D-4E57-9F93-75823784DC2A

for 1 minute at 4°C – to ensure submersion of biopsy in RNA*later*™ solution

4. Store this tumor sample at 4°C for maximal 1 month (or otherwise according to manufacturer's instructions)

9.1.2. Snap freezing of CNB

NOTE: Keep the CNB on ice while preparations

1. Switch on the horizontal laminar flow cabinet and clean thoroughly using RNase ZAP wipes
2. Weigh the 2mL Safe-Lock Eppendorf tube first and write down this weight
3. Lay down a KimWipe tissue on the surface of the horizontal laminar flow cabinet and transfer an RNase free disposable forceps on the KimWipe tissue
4. Place in the cabinet the 50mL centrifuge tube on ice and a sterile petri-dish
5. CNB is transferred from the 50mL centrifuge tube to the serile petri-dish using RNase free disposable forceps. In case CNB is longer than 1cm, biopsy is cut in two with sterile disposable scalpel to ease downstream homogenization
6. Tumor tissue pieces are transferred with the RNase free disposable forceps into a 2mL safe lock eppendorf tube – make sure the tissue pieces are at the bottom of the Eppendorf tube. Afterwards, weigh the 2mL safe lock eppendorf tube again and write down the weight. Calculate in the mean time the weight of the CNB, this will determine the volume RNA lysis buffer needed downstream.
7. Snap freeze the fresh CNB inserted in the 2mL safe lock eppendorf tube

NOTE: the CNB can be stored at -80°C (for at least 2 months).

9.2. Homogenization and lysis of FF core needle biopsy

9.2.1 Mechanical homogenization of FF core needle biopsy using the Tissuelyser II instrument

1. Insert the CNB in the horizontal laminar flow cabinet and add a 5mm stainless steel bead from the single-bead dispenser and close the 2mL safe lock Eppendorf tube.
2. Insert the 2mL safe lock Eppendorf tube, containing FF CNB and 5mm stainless steel bead, in the pre-cooled (-80°C) adapter set from the Tissuelyser II instrument.
3. Homogenize for 30 seconds at 30 hertz. In case not all tissue is homogenized, re-position the remaining tissue at the bottom of the 2mL safe lock Eppendorf tube in the horizontal laminar flow cabinet with the disposable RNase free forceps and repeat this step for a maximum total time of 2 minutes or 4 cycles.

9.2.2. Lysis of FF core needle biopsy using RLT buffer

1. After the mechanical homogenization step using the Tissuelyser II instrument, add RLT buffer (provided in the RNeasy kit from Qiagen) complemented with β-mercaptoethanol (β-ME) (10μL of β-ME/1mL RLT), according to Qiagen's recommendations (tissues <20mg=350μL, tissues ≤30mg=600μL). Place the 2mL safe lock Eppendorf tube with homogenized CNB and RLT buffer at 4°C overnight (ON) to incubate

3. After ON incubation at 4°C, transfer the 2mL safe lock Eppendorf tube and vortex during 1 hour at 4°C

9.2.3 Additional homogenization using the Tissueruptor II dissociator (in case the FF core needle biopsy is not fully disrupted)

1. In case the tissue is not fully disrupted, transfer the lysate and remaining tissue to a 50mL centrifuge tube and homogenize the remaining core needle tumor tissue using the Tissueruptor II dissociator – foaming can exist
2. Detach the probe and rinse the probe with an additional 350µL/600µL RLT buffer – depending on the initial volume used for lysis
3. Briefly spin the 50mL centrifuge tube down (375 rcf, 1minute, 4°C) using Eppendorf centrifuge

9.2.4 Total RNA extraction from lysate

Transfer the lysate to a 1.5mL Eppendorf DNA LoBind tube and proceed with the standard protocol of the Qiagen RNeasy kit

NOTE: To obtain a higher RNA yield the eluate was reloaded on the elution column

10. Annex

The Quick-Start Protocol to extract total RNA from (tumor) tissue using the RNeasy Mini kit from Qiagen is attached in annex.

DocuSign Envelope ID: 1986343D-604D-4E57-9F93-75823784DC2A

Quick-Start Protocol March 2016

RNeasy® Mini Kit, Part 1

The RNeasy Mini Kit (cat. nos. 74104 and 74106) can be stored at room temperature (15–25°C) for at least 9 months if not otherwise stated on label.

Further information

- *RNeasy Mini Handbook*: **www.qiagen.com/HB-0435**
- Safety Data Sheets: **www.qiagen.com/safety**
- Technical assistance: **support.qiagen.com**

Notes before starting

- If purifying RNA from cell lines rich in RNases, or tissue, add either 10 µl β-mercaptoethanol (β-ME), or 20 µl 2 M dithiothreitol (DTT),* to 1 ml Buffer RLT. Buffer RLT with β-ME or DTT can be stored at room temperature for up to 1 month.
- Add 4 volumes of ethanol (96–100%) to Buffer RPE for a working solution.
- Remove RNA*later*® stabilized tissue from the reagent using forceps.
- For RNeasy Protect Mini Kit (cat. nos. 74124 and 74126), please start with the *Quick-Start Protocol RNAlater RNA Stabilization Reagent, RNAlater TissueProtect Tubes, and RNeasy Protect Kits*.

* This option not included for cells in handbook; handbook to be updated.

1. **Cells**: Harvest a maximum of 1 x 10^7 cells, as a cell pellet or by direct lysis in the vessel. Add the appropriate volume of Buffer RLT (see Table 1).

   Tissues: Do not use more than 30 mg tissue. Disrupt the tissue and homogenize the lysate in the appropriate volume of Buffer RLT (see Table 1). Centrifuge the lysate for 3 min at maximum speed. Carefully remove the supernatant by pipetting, and use it in step 2.
2. Add 1 volume of 70% ethanol to the lysate, and mix well by pipetting. Do not centrifuge. Proceed immediately to step 3.
3. Transfer up to 700 µl of the sample, including any precipitate, to an RNeasy Mini spin column placed in a 2 ml collection tube (supplied). Close the lid, and centrifuge for 15 s at ≥8000 x *g*. Discard the flow-through.

   Optional: For DNase digestion, follow steps 1–4 of "On column DNase digestion" in *Quick-Start Protocol RNeasy Mini Kit, Part 2*.
4. Add 700 µl Buffer RW1 to the RNeasy spin column. Close the lid, and centrifuge for 15 s at ≥8000 x *g*. Discard the flow-through.
5. Add 500 µl Buffer RPE to the RNeasy spin column. Close the lid, and centrifuge for 15 s at ≥8000 x *g*. Discard the flow-through.
6. Add 500 µl Buffer RPE to the RNeasy spin column. Close the lid, and centrifuge for 2 min at ≥8000 x *g*.

   Optional: Place the RNeasy spin column in a new 2 ml collection tube (supplied). Centrifuge at full speed for 1 min to dry the membrane.
7. Place the RNeasy spin column in a new 1.5 ml collection tube (supplied). Add 30–50 µl RNase-free water directly to the spin column membrane. Close the lid, and centrifuge for 1 min at ≥8000 x *g* to elute the RNA.
8. If the expected RNA yield is >30 µg, repeat step 7 using another 30–50 µl of RNase-free water, or using the eluate from step 7 (if high RNA concentration is required). Reuse the collection tube from step 7.

Table 1. Volumes of Buffer RLT for sample disruption and homogenization

Sample	Amount	Dish	Buffer RLT	Disruption and homogenization
Animal cells	<5 x 10^6	<6 cm	350 µl	Add Buffer RLT, vortex (≤1 x 10^5 cells); or use QIAshredder, TissueRuptor®, or needle and syringe
	≤1 x 10^7	6–10 cm	600 µl	
Animal tissues	<20 mg	–	350 µl*	TissueLyser LT; TissueLyser II; TissueRuptor, or mortar and pestle followed by QIAshredder or needle and syringe
	≤30 mg	–	600 µl	

* Use 600 µl Buffer RLT for tissues stabilized in RNA*later*, or for difficult-to-lyse tissues.

Scan QR code for handbook.

For up-to-date licensing information and product-specific disclaimers, see the respective QIAGEN kit handbook or user manual. Trademarks: QIAGEN®, Sample to Insight®, RNeasy®, TissueRuptor® (QIAGEN Group). "RNA*later*®" is a trademark of AMBION, Inc., Austin, Texas and is covered by various U.S. and foreign patents.
1101297 03/2016 HB-0570-002 © 2016 QIAGEN, all rights reserved.

DocuSign Envelope ID: 1986343D-604D-4E57-9F93-75823784DC2A

Quick-Start Protocol April 2016

RNeasy® Mini Kit, Part 2

The RNeasy Mini Kit (cat. nos. 74104 and 74106) can be stored at room temperature (15–25°C) for at least 9 months if not otherwise stated on label.

Further information

- *RNeasy Mini Handbook*: **www.qiagen.com/HB-0435**
- Safety Data Sheets: **www.qiagen.com/safety**
- Technical assistance: **support.qiagen.com**

Notes before starting

On-column DNase digestion

- If using the RNase-Free DNase Set for the first time, prepare DNase I stock solution by injecting 550 µl RNase-free water into the DNase I vial using an RNase-free needle and syringe. Mix gently by inverting the vial. Do not vortex.
- For long-term storage of DNase I stock solution, divide it into single-use aliquots and store at –20°C for up to 9 months. Thawed aliquots can be stored at 2–8°C for up to 6 weeks. Do not refreeze aliquots after thawing.

1. Add 350 µl Buffer RW1 to RNeasy column, close lid, centrifuge for 15 s at ≥8000 x *g* (≥10,000 rpm). Discard flow-through.
2. Add 10 µl DNase I stock solution (see above) to 70 µl Buffer RDD. Mix by gently inverting the tube. Centrifuge briefly.
3. Add DNase I incubation mix (80 µl) directly to RNeasy column membrane, and place on benchtop (20–30°C) for 15 min.
4. Add 350 µl Buffer RW1 to RNeasy column, close lid, centrifuge for 15 s at ≥8000 x *g*. Discard flow-through. Continue with step 5 of "RNA purification from cells/tissue samples" in *Quick-Start Protocol RNeasy Mini Kit, Part 1*, or step 4 of "RNA cleanup" (below).

Sample to Insight

QIAGEN

Notes before starting

RNA cleanup

- Add 4 volumes of ethanol (96–100%) to Buffer RPE for a working solution.

1. Adjust the sample to a volume of 100 µl with RNase-free water. Alternatively, follow steps in "DNase digestion of RNA before RNA cleanup" in Appendix E of *RNeasy Mini Handbook*. Add 350 µl Buffer RLT, and mix well.
2. Add 250 µl ethanol (96–100%) to the diluted RNA, and mix well by pipetting. Do not centrifuge. Proceed immediately to step 3.
3. Transfer the sample (700 µl) to an RNeasy Mini spin column placed in a 2 ml collection tube (supplied). Close the lid. Centrifuge for 15 s at ≥8000 x *g*. Discard the flow-through.

   Optional: If performing optional on-column DNase digestion, follow steps 1–4 of "On column DNase digestion" (above) after this step.
4. Add 500 µl Buffer RPE to the RNeasy spin column. Close the lid. Centrifuge for 15 s at ≥8000 x *g* to wash the membrane. Discard the flow-through.
5. Add 500 µl Buffer RPE to the RNeasy spin column. Close the lid. Centrifuge for 2 min at ≥8000 x *g* to wash the membrane.

   Optional: Place the RNeasy spin column in a new 2 ml collection tube (supplied). Close the lid, and centrifuge at full speed for 1 min.
6. Place the RNeasy spin column in a new 1.5 ml collection tube (supplied). Add 30–50 µl RNase-free water directly to the spin column membrane. Close the lid, and centrifuge for 1 min at ≥8000 x *g* to elute the RNA.
7. If the expected RNA yield is >30 µg, repeat step 6 using another 30–50 µl of RNase-free water. Alternatively, use the eluate from step 6 (if high RNA concentration is required). Reuse the collection tube from step 6.

Scan QR code for handbook.

For up-to-date licensing information and product-specific disclaimers, see the respective QIAGEN kit handbook or user manual. Trademarks: QIAGEN®, RNeasy®, Sample to Insight® (QIAGEN Group). 1102231 04/2016 HB-0571-002 © 2016 QIAGEN, all rights reserved.
